# Association of Parental Status and Gender With Burden of Multidisciplinary Tumor Boards Among Oncology Physicians

**DOI:** 10.1001/jamanetworkopen.2023.40663

**Published:** 2023-10-31

**Authors:** Brittney L. Chau, Jonnby S. LaGuardia, Sungjin Kim, Samuel C. Zhang, Eric Pletcher, Nina N. Sanford, Ann C. Raldow, Lisa Singer, Jun Gong, Sukhmani K. Padda, Mitchell Kamrava, Tara Cohen, Devarati Mitra, Katelyn M. Atkins

**Affiliations:** 1 Department of Medicine, New York Medical College, New York, New York; 2 Department of Medicine, University of Rochester Medical Center, Rochester, New York; 3 Biostatistics Research Center, Cedars-Sinai Medical Center, Los Angeles, California; 4 Department of Radiation Oncology, Cedars-Sinai Medical Center, Los Angeles, California; 5 Department of Surgery, Cedars-Sinai Medical Center, Los Angeles, California; 6 Department of Radiation Oncology, University of Texas Southwestern Medical Center, Dallas; 7 Department of Radiation Oncology, University of California Los Angeles Medical Center, Los Angeles; 8 Department of Radiation Oncology, University of California San Francisco Medical Center, San Francisco; 9 Department of Medicine, Division of Medical Oncology, Cedars-Sinai Medical Center, Los Angeles, California; 10 Department of Radiation Oncology, MD Anderson Cancer Center, Houston, Texas

## Abstract

**Importance:**

Tumor boards are integral to the care of patients with cancer. However, data investigating the burden of tumor boards on physicians are limited.

**Objective:**

To investigate what physician-related and tumor board–related factors are associated with higher tumor board burden among oncology physicians.

**Design, Setting, and Participants:**

Tumor board burden was assessed by a cross-sectional convenience survey posted on social media and by email to Cedars-Sinai Medical Center cancer physicians between March 3 and April 3, 2022. Tumor board start times were independently collected by email from 22 top cancer centers.

**Main Outcomes and Measures:**

Tumor board burden was measured on a 4-point scale (1, not at all burdensome; 2, slightly burdensome; 3, moderately burdensome; and 4, very burdensome). Univariable and multivariable probabilistic index (PI) models were performed.

**Results:**

Surveys were completed by 111 physicians (median age, 42 years [IQR, 36-50 years]; 58 women [52.3%]; 60 non-Hispanic White [54.1%]). On multivariable analysis, factors associated with higher probability of tumor board burden included radiology or pathology specialty (PI, 0.68; 95% CI, 0.54-0.79; *P* = .02), attending 3 or more hours per week of tumor boards (PI, 0.68; 95% CI, 0.58-0.76; *P* < .001), and having 2 or more children (PI, 0.65; 95% CI, 0.52-0.77; *P* = .03). Early or late tumor boards (before 8 am or at 5 pm or after) were considered very burdensome by 33 respondents (29.7%). Parents frequently reported a negative burden on childcare (43 of 77 [55.8%]) and family dynamics (49 of 77 [63.6%]). On multivariable analysis, a higher level of burden from early or late tumor boards was independently associated with identifying as a woman (PI, 0.69; 95% CI, 0.57-0.78; *P* = .003) and having children (PI, 0.75; 95% CI, 0.62-0.84; *P* < .001). Independent assessment of 358 tumor boards from 22 institutions revealed the most common start time was before 8 am (88 [24.6%]).

**Conclusions and Relevance:**

This survey study of tumor board burden suggests that identifying as a woman or parent was independently associated with a higher level of burden from early or late tumor boards. The burden of early or late tumor boards on childcare and family dynamics was commonly reported by parents. Having 2 or more children, attending 3 or more hours per week of tumor boards, and radiology or pathology specialty were associated with a significantly higher tumor board burden overall. Future strategies should aim to decrease the disparate burden on parents and women.

## Key Points

**Question:**

What is the burden of tumor boards among oncology physicians?

**Findings:**

In this survey study of tumor board burden, identifying as a woman or a parent was associated with a significantly higher level of burden from early morning and/or late evening tumor boards. Having 2 or more children, attending 3 or more hours per week of tumor boards, or being a radiologist or pathologist was associated with a significantly higher level of burden overall.

**Meaning:**

This study suggests that future strategies should aim to decrease tumor board burden, particularly the disparate burden on parents, women, and/or specific specialists.

## Teaser

This survey study investigates what physician-related and tumor board–related factors are associated with higher tumor board burden among oncology physicians.

## Introduction

Tumor boards are multidisciplinary team meetings focused on clinical assessment and decision-making in cancer care. They are a criterion standard in oncologic practice and a key part of clinical practice guidelines.^1^ Tumor board discussions often result in changes in diagnosis and treatment, underscoring the complexity of caring for patients with cancer.^1,2,3^ Given the specialized expertise tumor boards require, there can be significant logistical and administrative challenges in bringing together representatives from all disciplines.^1^

Significant time and effort are spent in the preparation and running of tumor boards.^3^ Tumor boards are often held weekly or biweekly, and many physicians, especially those who treat multiple disease sites, may attend several tumor boards per week. This commitment can include time for both preparation and attendance, particularly if they are leaders of the tumor board and/or responsible for preparing the data, as well as commuting for in-person participation. Several obstacles to tumor board effectiveness have been reported, including poor attendance due to time constraints, unprotected meeting times, and lack of administrative support and funding.^3^ Moreover, tumor board meetings may variably be held at early and/or late hours. However, data describing this practice are lacking.

Although there is growing research investigating the association between parenthood and physician work-life balance^4,5^; the contribution of increased, uncompensated administrative burden or excessive clerical work; and work-home conflicts with physician burnout,^6^ there is a paucity of data evaluating tumor board–associated burden among oncology physicians. We hypothesize that the burden of tumor boards is readily detectable and that early morning and/or late evening tumor board meetings may be particularly burdensome, especially for physicians who are parents or who have additional demands from their personal lives. Therefore, in this study, we aim to measure the overall burden of tumor boards as well as the burden of early morning and/or late evening tumor board meetings, with the goal of identifying factors associated with greater physician-assessed burden.

## Methods

### Survey Formation

We developed an original survey instrument (eAppendix in Supplement 1) following the American Association for Public Opinion Research (AAPOR) guideline and checklist for reporting results of internet e-surveys.^7^ This survey instrument was not validated. This cross-sectional survey study was approved by the Cedars-Sinai Medical Center institutional review board. Participants provided written informed consent.

### Survey Collection

The study population was drawn from practicing physicians involved in oncology care from hospitals and medical centers throughout the US. Participants were recruited by either (1) social media links posted on Twitter and Facebook, including Facebook groups for physician parents, or (2) email to Cedars-Sinai oncology physicians via listservs. Participants were invited to participate in a voluntary, anonymous survey. The survey was open to participation from March 3 through April 3, 2022. Participants were informed that the survey’s purpose was to better understand the administrative burden of multidisciplinary tumor boards. Respondents were provided an information sheet to review, and on agreeing to the terms and providing written informed consent, participants completed the survey via REDCap (Vanderbilt University) (REDCap survey instrument in eAppendix in Supplement 1). The survey was not pretested or validated. Only completed surveys were analyzed.

### Tumor Board Start Times

Tumor board start times were independently collected (ie, not assessed by the REDCap survey instrument). Personal colleagues of the coauthors in radiation or medical oncology at National Cancer Institute (NCI)–designated cancer centers or *US World and News Report* top 40 hospitals for cancer were contacted individually by email from November 16, 2021, through January 3, 2022, to determine the start times of regularly scheduled multidisciplinary tumor board meetings. Early morning tumor board meeting start times were defined as occurring before 8 am, while late evening tumor board meeting were defined as occurring at 5 pm or after.

### Statistical Analysis

Data are presented as the number and percentage of respondents for categorical variables and as mean (SD) or median (IQR) values for continuous variables. The primary outcomes were the level of overall tumor board burden (TBB) and early or late TBB as measured on a scale of 1 to 4 (1, not at all burdensome; 2, slightly burdensome; 3, moderately burdensome; and 4, very burdensome) as reported by respondents.

Univariable and multivariable analyses of outcomes were carried out using the probabilistic index (PI) model,^8,9^ where the PI is a measure associated with the Mann-Whitney test. The probability that an outcome value for one group is greater than or equal to the outcome value for another group was estimated, along with a Wald-type 95% CI. *P* values were calculated using Wald statistics. In PI model analyses, covariates with *P* < .10 on univariate analysis were included in multivariable analyses. In the multivariable analyses, multicollinearity was assessed by the variance inflation factor. Model fit was assessed as previously described.^10^ Analyses were performed using R, version 4.0.5 (R Project for Statistical Computing),^11^ with 2-sided tests at a significance level of *P* < .05. Respondents’ comments were visually summarized using word clouds, and inductive thematic analysis was performed. Analyses were performed using R, version 4.0.5 (*pim, tm,* and *wordcloud* libraries).

## Results

### Characteristics of Respondents

In total, there were 111 complete surveys (of 117 initiated). The median age of respondents was 42 years (IQR, 36-50 years), with 52.3% of participants (n = 58) identifying as women and 42.3% (n = 47) as men (Table 1). Radiation oncology was the most common specialty reported (39.6% [n = 44]), although respondents came from all specialties, including medical oncology, surgery, radiology, and pathology. Although more than half of participants (56.8% [n = 63]) attended 1 to 2 hours of tumor boards per week, 41.4% (n = 46) attended 3 or more hours of tumor boards per week, and 58.6% (n = 65) attended 1 to 2 hours per week of early or late tumor boards (defined as starting before before 8 am or at 5 pm or after). In addition to tumor boards, 55.0% of participants (n = 61) spent an additional 1 to 2 hours and 27.9% of participants (n = 31) an additional 3 or more hours per week on other departmental or multidisciplinary rounds, such as medical record rounds or contour rounds. Furthermore, nearly all respondents reported additional hours per week on other administrative work or meetings, with 29.7% (n = 33) spending 1 to 2 hours, 24.3% (n = 27) 3 to 4 hours, 19.8% (n = 22) 5 to 6 hours, and 24.3% (n = 27) more than 6 hours weekly on other administrative work or meetings. Only 8 respondents (7.2%) were provided any form of compensation for time attending tumor boards. Of these, the compensation types were monetary for 4 respondents, with 2 respondents each receiving relative value unit–based compensation or compensation built into salary expectations. Further demographic, employment, and administrative characteristic data from respondents are summarized in Table 1.

**Table 1.** Demographic and Employment Characteristics

Variable	No. of respondents (%) (N = 111)
Age, median (IQR), y	42 (36-50)
Gender	
Man	47 (42.3)
Woman	58 (52.3)
Prefer not to say or missing	6 (5.4)
Race and ethnicity	
Hispanic or Latinx/a/o/e	3 (2.7)
Non-Hispanic Asian	31 (27.9)
Non-Hispanic Black or African American	3 (2.7)
Non-Hispanic Middle Eastern or North African	4 (3.6)
Non-Hispanic White	60 (54.1)
Prefer not to say or missing	10 (9.0)
Medical specialty	
Radiation oncology	44 (39.6)
Medical oncology	20 (18.0)
Radiology	14 (12.6)
Pathology	11 (9.9)
Surgical oncology or surgical subspecialty	17 (15.3)
Other or multiple	5 (4.5)
Years in practice	
≤5	36 (32.4)
6-10	21 (18.9)
11-15	15 (13.5)
>15	34 (30.6)
NA: resident or fellow	5 (4.5)
Employment institution type	
Academic medical center or affiliate or network site or NCI-designated Comprehensive Cancer Center	82 (73.9)
Community hospital, hospital based, or private practice	19 (17.1)
Multiple	10 (9.0)
No. of disease sites treated	
1-2	65 (58.6)
3-4	18 (16.2)
>4	28 (25.2)
Mean tumor board h/wk	
1-2	63 (56.8)
3-4	35 (31.5)
5-6	8 (7.2)
>6	3 (2.7)
0	2 (1.8)
Mean h/wk of early or late tumor boards (<8 am or ≥5 pm)	
1-2	65 (58.6)
3-4	9 (8.1)
5-6	2 (1.8)
>6	1 (0.9)
0	34 (30.6)
Mean additional h/wk in other departmental or multidisciplinary rounds	
1-2	61 (55.0)
3-4	19 (17.1)
5-6	7 (6.3)
>6	5 (4.5)
0	19 (17.1)
Mean additional h/wk spent on other administrative work or in meetings	
1-2	33 (29.7)
3-4	27 (24.3)
5-6	22 (19.8)
>6	27 (24.3)
0	2 (1.8)
Compensated for time in tumor board	
No	103 (92.8)
Yes	8 (7.2)

Abbreviations: NA, not applicable; NCI, National Cancer Institute.

### Tumor Board Burden

Nearly half (49.5% [n = 55]) of respondents agreed or strongly agreed that the number of tumor boards they attended was burdensome. Overall, 37.8% of respondents (n = 42) reported that tumor boards were moderately (28.8% [n = 32]) or very burdensome (9.0% [n = 10]), while only 9.9% (n = 11) reported that tumor boards were not at all burdensome (Table 2). When asked if the number of tumor board meetings attended contributes to their professional burnout, 42.3% of respondents (n = 47) agreed, and 12.6% (n = 14) strongly agreed. Nearly all respondents agreed (33.3% [n = 37]) or strongly agreed (57.7% [n = 64]) that the transition to virtual tumor boards during the SARS-CoV-2 pandemic has made attending tumor boards easier and would prefer that tumor boards remain virtual in the future (18.9% [n = 21] agree and 51.4% [n = 57] strongly agree). Furthermore, nearly all respondents (94.6% [n = 105]) indicated that 76% to 100% of boards have remained virtual since the pandemic transition.

**Table 2.** Burden Level and Home Life Factor Characteristics

Variable	No. of respondents (%) (N = 111)
Children	
No	34 (30.6)
Yes	77 (69.4)
No. of children^a^	
1	47/77 (61.0)
≥2	30/77 (39.0)
Age of youngest child, y^a^	
≤2	21/77 (27.3)
3-5	16/77 (20.8)
6-12	19/77 (24.7)
≥13	21/77 (27.3)
Age of oldest child, y^a^	
≤2	12/77 (15.6)
3-5	11/77 (14.3)
6-12	22/77 (28.6)
≥13	32/77 (41.6)
Do early or late tumor boards (<8 am or ≥5 pm) negatively affect your childcare logistics?^a^	
No	13/77 (16.9)
Yes	43/77 (55.8)
Not applicable	21/77 (27.3)
Do early or late tumor boards (<8 am or ≥5 pm) negatively affect your child or children feeding and/or sleep logistics?^a^	
No	26/77 (33.8)
Yes	26/77 (33.8)
Not applicable	25/77 (32.5)
Overall tumor board burden	
Not at all burdensome (0)	11 (9.9)
Slightly burdensome (1)	58 (52.3)
Moderately burdensome (2)	32 (28.8)
Very burdensome (3)	10 (9.0)
Early or late tumor board burden	
Not at all burdensome (0)	5 (4.5)
Slightly burdensome (1)	25 (22.5)
Moderately burdensome (2)	23 (20.7)
Very burdensome (3)	33 (29.7)
Not applicable	25 (22.5)

^a^ Includes responses only from those with children.

On multivariable analysis, radiology or pathology specialty (PI, 0.68; 95% CI, 0.54-0.79; *P* = .02), attending 3 or more hours per week of tumor boards (PI, 0.68; 95% CI, 0.58-0.76; *P* < .001), and having 2 or more children (PI, 0.65; 95% CI, 0.52-0.77; *P* = .03) were associated with a higher level of burden after adjusting for number of primary treating disease sites and compensation (Table 3).

**Table 3.** Univariate and Multivariable Analyses of Level of Overall Tumor Board Burden^a^

Comparison A < B^b^	Univariate		Multivariable	
	PI (95% CI)^c^	*P* value	PI (95% CI)^c^	*P* value
Age (1-y increment)	0.50 (0.50-0.51)	.39	Not included^d^	NA
Gender				
Man	[Reference]	NA	NA	NA
Woman	0.54 (0.44-0.64)	.40	Not included^d^	NA
Prefer not to say or missing	0.46 (0.27-0.67)	.74		
Race and ethnicity				
Non-Hispanic White	[Reference]	NA	NA	NA
Hispanic or non-Hispanic Black or African American or non-Hipanic Middle Eastern or North African	0.38 (0.23-0.56)	.19	Not included^d^	NA
Non-Hispanic Asian	0.47 (0.36-0.59)	.63		
Prefer not to say or missing	0.52 (0.35-0.67)	.87		
What is your specialty?				
Radiation oncology	[Reference]	NA	NA	NA
Medical oncology	0.51 (0.37-0.65)	.90	0.57 (0.42-0.70)	.35
Radiology or pathology	0.60 (0.47-0.72)	.13	0.68 (0.54-0.79)	.02
Surgical or other	0.31 (0.21-0.43)	.003	0.39 (0.28-0.51)	.08
Multiple	0.66 (0.22-0.93)	.51	0.74 (0.27-0.96)	.33
How many years have you been in practice?				
>15 y	[Reference]	NA	NA	NA
Resident or fellow or ≤5 y	0.48 (0.36-0.60)	.71	Not included^d^	NA
6-15 y	0.52 (0.40-0.64)	.71		
How many years have you been in practice? (binary)				
>15 y	[Reference]	NA	NA	NA
Resident or fellow or ≤15 y	0.50 (0.39-0.61)	.98	Not included^d^	NA
What type of institution do you work at? (binary)				
Community hospital or hospital-based practice or private or group practice	[Reference]	NA	NA	NA
Academic medical center or academic medical center affiliate or network site or NCI-designated Comprehensive Cancer Center	0.45 (0.32-0.58)	.42	Not included^d^	NA
Multiple	0.45 (0.27-0.63)	.58		
How many disease sites do you primarily treat? (binary)				
1-2	[Reference]	NA	NA	NA
≥3	0.6 (0.52-0.71)	.02	0.58 (0.47-0.68)	.17
How many total hours of tumor boards do you attend on an average week? (binary)				
0-2	[Reference]	NA	NA	NA
≥3	0.65 (0.58-0.74)	.002	0.68 (0.58-0.76)	<.001
How many hours of tumor boards are before 8 am or after 5 pm on an average week? (binary)				
0-2	[Reference]	NA	NA	NA
≥3	0.59 (0.45-0.71)	.21	Not included^d^	NA
How many additional hours are spent on other departmental or multidisciplinary rounds on an average week? (binary)				
0-2	[Reference]	NA	NA	NA
≥3	0.55 (0.44-0.65)	.36	Not included^d^	NA
How many hours are spent on other administrative work or in meetings on an average week? (binary)				
0-4	[Reference]	NA	NA	NA
≥5	0.49 (0.40-0.59)	.87	Not included^d^	NA
Are you compensated for your time in tumor boards?				
No	[Reference]	NA	NA	NA
Yes	0.70 (0.54-0.83)	.01	0.65 (0.47-0.80)	.10
Do you have children?				
No	[Reference]	NA	NA	NA
Yes	0.58 (0.47-0.68)	.17	Not included^d^	NA
No. of children				
0	[Reference]	NA	NA	NA
1	0.55 (0.43-0.66)	.46	0.58 (0.46-0.69)	.22
≥2	0.62 (0.50 0.74)	.06	0.65 (0.52-0.77)	.03

Abbreviations: NA, not applicable; NCI, National Cancer Institute; PI, probabilistic index.

^a^ There were 111 observations used in the multivariable model. If PI is greater than 0.5, then the probability that the overall tumor board burden for B is greater than A is high, indicating that B has higher overall tumor board burden. If the PI is less than 0.5, then the probability that the overall tumor board burden for B is greater than A is low, indicating that A has higher overall tumor board burden.

^b^ Comparison denotes the probability that overall tumor board burden for B is higher than that for A (reference level).

^c^ A PI of 0.5 indicates no difference between comparisons (ie, A = B).

^d^ Not included in the multivariable model.

For early or late (before 8 am or at 5 pm or after) TBB, more than half (50.5% [n = 56]) of respondents reported that early or late tumor boards were moderately (20.7% [n = 23]) or very burdensome (29.7% [n = 33]), while only 4.5% (n = 5) reported that early or late tumor boards were not at all burdensome (Table 2). On multivariable analysis, identifying as a woman (PI, 0.69; 95% CI, 0.57-0.78; *P* = .003) and having children (PI, 0.75; 95% CI, 0.62-0.84; *P* < .001) remained independently associated with a higher level of early or late TBB (Table 4).

**Table 4.** Univariate and Multivariable Analyses of Level of Early or Late Tumor Board Burden^a^

Comparison A < B^b^	Univariate		Multivariable^c^	
	PI (95% CI)^d^	*P* value	PI (95% CI)^d^	*P* value
Age (1-y increment)	0.50 (0.49-0.51)	.88	Not included^e^	NA
Gender^f^				
Man	[Reference]	NA	NA	NA
Woman	0.63 (0.52-0.74)	.03	0.69 (0.57-0.78)	.003
Race and ethnicity				
Non-Hipanic White	[Reference]	NA	NA	NA
Hispanic or non-Hispanic Black or African American or non-Hispanic Middle Eastern or North African	0.44 (0.260-0.63)	.54	Not included^e^	NA
Non-Hispanic Asian	0.50 (0.36-0.64)	≥.99		
Prefer not to say or missing	0.59 (0.44-0.73)	.23		
What is your specialty?				
Radiation oncology	[Reference]	NA	NA	NA
Medical oncology	0.40 (0.25-0.57)	.24	Not included^e^	NA
Radiology or pathology	0.48 (0.33-0.64)	.83		
Surgical or other	0.38 (0.24-0.54)	.14		
Multiple	0.61 (0.36-0.81)	.40		
How many years have you been in practice?				
>15 y	[Reference]	NA	NA	NA
Resident or fellow or ≥5 y	0.50 (0.36-0.64)	.96	Not included^e^	NA
6-15 y	0.42 (0.28-0.58)	.34		
How many years have you been in practice? (binary)				
>15 y	[Reference]	NA	NA	NA
Resident or fellow or ≤15 y	0.47 (0.34-0.60)	.62	Not included^e^	NA
What type of institution do you work at? (binary)				
Community hospital or hospital-based practice or private or group practice	[Reference]	NA	NA	NA
Academic medical center or academic medical center affiliate or network site or NCI-designated Comprehensive Cancer Center	0.53 (0.36-0.70)	.72	Not included^e^	NA
Multiple	0.48 (0.26-0.71)	.86		
How many disease sites do you primarily treat? (binary)				
1-2	[Reference]	NA	NA	NA
≥3	0.51 (0.39-0.62)	.94	Not included^e^	NA
How many total hours of tumor boards do you attend on an average week? (binary)				
0-2	[Reference]	NA	NA	NA
≥3	0.58 (0.46-0.69)	.18	Not included^e^	NA
How many hours of tumor boards are before 8 am or after 5 pm on an average week? (binary)				
0-2	[Reference]	NA	NA	NA
≥3	0.46 (0.29-0.64)	.66	Not included^e^	NA
How many additional hours are spent on other departmental or multidisciplinary rounds on an average week? (binary)				
0-2	[Reference]	NA	NA	NA
≥3	0.45 (0.32-0.57)	.40	Not included^e^	NA
How many hours are spent on other administrative work or in meetings on an average week? (binary)				
0-4	[Reference]	NA	NA	NA
≥5r	0.48 (0.36-0.59)	.67	Not included^e^	NA
Are you compensated for your time in tumor boards?				
No	[Reference]	NA	NA	NA
Yes	0.63 (0.40-0.81)	.26	Not included^e^	NA
Do you have children?				
No	[Reference]	NA	NA	NA
Yes	0.69 (0.56-0.79)	.003	0.75 (0.62-0.84)	<.001

Abbreviations: NA, not applicable; NCI, National Cancer Institute; PI, probabilistic index.

^a^ If PI is greater than 0.5, then probability that early or late tumor board burden for B is greater than A is high, indicating that B has higher early or late tumor board burden. If PI is less than 0.5, then probability that early or late tumor board burden for B is greater than A is low, indicating that A has higher early or late tumor board burden.

^b^ Comparison A < B denotes the probability that early or late tumor board burden for B is higher than that for A (reference level).

^c^ A total of 84 observations were used in the multivariable model.

^d^ A PI of 0.5 indicates no difference between comparisons (ie, A = B).

^e^ Not included in the multivariable model.

^f^ Two participants who responded “prefer not to answer” were excluded from analyses.

Most respondents (69.4% [n = 77]) had children (61.0% [47 of 77] with 1 child and 39.0% [30 of 77] with ≥2 children) (Table 2). Early or late tumor boards were negatively associated with childcare logistics (eg, nanny and day care) for more than half of respondents (55.8% [43 of 77]) and feeding (including direct breastfeeding and/or pumping) and/or sleep logistics for one-third of respondents with children (33.8% [26 of 77]). Similarly, parents agreed (32.5% [25 of 77]) or strongly agreed (31.2% [24 of 77]) that early or late tumor boards were negatively associated with their family dynamics overall.

### Qualitative Analysis of Respondent Comments

More than one-fourth of respondents (28.8% [n = 32]) provided comments. Thematic analysis of general respondent comments revealed 16 subthemes among 5 primary themes encompassing value or compensation (n = 10), feedback or gratitude (n = 10), administrative or scheduling (n = 7), wellness (n = 5), and family dynamics (n = 4) (Box). Specifically, 4 participants commented that their time preparing for the tumor boards was not valued, 3 participants noted that their time commitment in general was not valued, and 3 emphasized that their time was uncompensated, including 1 participant stating that they spend 3 to 6 hours preparing for tumor boards each week, “all uncompensated and unrecognized time.” Five participants commented on the demand of early or late tumor boards or other meetings affecting their wellness and work-life balance, with 1 participant stating, “the main issue with meetings before 0800 or after 1700 is the impact [on their] ability to engage in [their] wellness activities, which has aided in prevent[ing] burnout.” Two respondents commented specifically that recurring administrative meetings and tumor boards should not be before 0800 or after 1700, with 1 participant adding, “especially as these clinical activities are not compensated despite being critically important in the management of patient care.” Five participants expressed gratitude for the study, with 1 participant commenting that the study was “asking the real questions for the future.”

Box. **Thematic Analysis of Respondent Comments**

**Primary theme**

Administrative or scheduling

**Subtheme**

Difficulty of dual physician partners for scheduling (n = 1)
Ease of virtual meetings has created more meetings (n = 3)
High volume of other administrative burdens (n = 1)
Virtual format makes it feasible to attend (n = 2)

**Primary theme**

Family dynamics

**Subtheme**

Burden on spouse (n = 2)
Early or late tumor board times are prohibitive due to childcare (n = 1)
Late tumor boards require multitasking if home (n = 1)

**Primary theme**

Feedback or gratitude

**Subtheme**

Expressed gratitude for study (n = 5)
Provided feedback for future work (n = 5)

**Primary theme**

Value or compensation

**Subtheme**

Specialist preparation time not valued (n = 4)
Time commitment is not valued (n = 3)
Time not compensated (n = 3)

**Primary theme**

Wellness

**Subtheme**

Disrupts wellness activities (n = 1)
Disrupts work-life balance (n = 1)
Dissatisfaction with meetings at lunch (n = 1)
No meetings outside regular business hours (n = 2)

Among 77 respondents who were parents, 18 (23.4%) provided comments about the association of early or late tumor boards with their children or family logistics. Thematic analysis revealed 6 themes, including disruption of transportation and/or nanny or day care logistics (n = 12), displaced burden to spouse or partner (n = 6), disruption of mealtime or feeding or breastfeeding (n = 4), requirement for multitasking (for virtual tumor boards) (n = 3), disruption of bedtime (n = 2), and less time with children (n = 2). Specifically, regarding disruption of transportation and/or nanny or day care logistics, participants noted, “it is impossible to juggle,” “makes day care drop-off impossible,” “I can’t pick up my kids,” “hard to get childcare,” or they must “carefully coordinate with partner’s schedule.” Within the theme of displaced burden to spouse or partner, 1 participant noted, “I’m unable to help. My wife manages drop-off and pickup,” another that “my wife does all the work,” while another commented that “it’s an ‘ask’ of my husband that ‘dings’ our relationship and the balance of ‘asks/gives.’” Word clouds summarizing the effect of tumor boards on childcare and feeding and sleep are shown in eFigure 1 in Supplement 1.

### Independent Assessment of Tumor Board Start Times

To independently assess the frequency of early or late tumor board occurrence, the times of 358 tumor boards were collected by email correspondence from 22 NCI-designated cancer centers and/or *US World and News Report* Top 40 hospitals for cancer **(**eFigure 2 and eTable in Supplement 1). The most common tumor board start time, when evaluated in 30-minute increments from 7:30 am or prior to 5 pm, was prior to 8 am (n = 88 [24.6%]), followed by 12 pm (n = 63 [17.6%]), and 5 pm or later (n = 34 [9.5%]). The mean (SD) duration of early or late tumor boards was 1.1 (0.3) hours.

## Discussion

In this survey study of TBB, identifying as a woman or parent was independently associated with increased burden from early or late tumor boards. Having 2 or more children, attending 3 or more hours per week of tumor boards, and radiology or pathology specialty were associated with a higher level of overall burden. Future strategies should aim to decrease the disparate burden on parents and women. Together, these results expand on prior research and add to the growing body of literature on workplace burden, physician wellness, and the burden on physician parents.

Several studies have shown that tumor boards are beneficial for patient management.^1,12,13^ This benefit requires multidisciplinary input because significant changes in management often occur from pathology, radiology, staging, and surgical clarifications.^14,15^ These findings are similarly observed at the global level, with a survey of oncologists from various Middle Eastern countries reporting that nearly all (93%) respondents agreed that tumor boards should be mandatory.^16^ Furthermore, in a survey distributed to international American Society of Clinical Oncology members, nearly all respondents reported that tumor boards provided an overall benefit to patients that was worth the time and effort. However, respondents also commonly wanted increased tumor board efficiency and a coordinator for case preparation.^17^ For pathologists and radiologists, considerable time is required to prepare cases (eg, review slides, microscopy, and images). Therefore, it is not surprising that radiology or pathology specialty was associated with a higher level of TBB in this study.

Our study observed that attending 3 or more hours per week of tumor board was associated with a higher level of burden. Furthermore, 42.3% agreed that the number of tumor boards attended was associated with their professional burnout. Although this finding does not directly support an association between tumor board hours and burnout, it is consistent with data reporting that increased work hours, clerical burden, and stressful professional events have been associated with burnout in oncologists.^18^ Similarly, burnout has been associated with oncologists’ decreasing sense of autonomy as administrative activities begin to outweigh direct patient care.^18,19,20^ Furthermore, several respondents in this study commented about the association of tumor boards with their wellness and wellness activities. And while individual-focused interventions, such as resiliency training or self-care programs, have been shown to help with burnout, institution-based strategies that call for wider organizational change have demonstrated a significant reduction in burnout.^21^ The combination of individual and institutional interventions is more effective, with longer-lasting results than person-directed interventions.^19,22,23^ Further investigation of burnout, depersonalization, and derealization would allow for a deeper understanding of the association between TBB and burnout and is warranted.

Our finding that women and parents reported increased TBB from early or late tumor boards and commonly reported a negative association with family dynamics is consistent with growing data describing the challenges of physician parents striving for work-life balance. Work-home conflict is more prevalent among physicians aged 35 to 44 years and among those with long work hours^24^ and is associated with burnout.^25,26^ Furthermore, a survey of women oncologists reported that the biggest sacrifice of an academic career was “time with loved ones.”^27^ Related to this finding, another survey study of female oncologists revealed that two-thirds described long work hours and heavy workload as negatively associated with family planning, while one-third experienced pregnancy or maternity leave–related discrimination.^28^ Another survey of predominantly female physicians revealed that 56% regretted delay of childbearing, while those with fertility challenges were more likely to report worse well-being, greater likelihood of relationship strain, and greater use of therapy associated with family building.^29^ Additional barriers for women physicians include challenges associated with lactation and breastfeeding after a return to work, lack of part-time work opportunities, and difficulties finding childcare.^30^ A survey of female physicians found that while most (>75%) would like childcare options through their workplace, only 7% had such access.^5^

Several solutions are proposed, including organizational changes that prioritize physician wellness and reduce administrative burden, as well as reducing or eliminating meetings (such as tumor boards) at early or late times and continuing tumor boards in a virtual format.^31^ Another consideration would be a novel redesign in favor of an online, “asynchronous” tumor board, where participating physicians would not need to be logged on at the same time but could create threads and direct questions toward specific specialties. Although data reporting on asynchronous tumor boards are limited, initial studies have reported the development of scalable cloud-based virtual molecular tumor boards that incorporate asynchronous chats^32^ and remote, asynchronous subspecialist input, such as the AccessHope program.^33^ Other considerations based on popular use are crowdsourced expertise or online novel tools, such as theMednet^34^ or CIViC—a crowdsourced knowledge base for clinical interpretation of genetic variants in cancer.^35^ Last, 7.2% of physicians reported receiving compensation for tumor board time. Thus, another solution could be implementation of monetary or relative value unit–based compensation, or “time banking” for uncompensated service and/or administrative activities that can translate to tangible benefits. One example of this is a time bank piloted by Stanford University, which allowed faculty to trade time spent on usually uncompensated activities, such as serving on committees, for in-home support, such as meal delivery or cleaning services.^36^ These changes corresponded to a doubling of the number of women faculty members who felt supported in their career development.

### Limitations

This study has several limitations. First, social media was used to distribute a targeted convenience survey; thus, we were unable to assess response rate or characterize nonresponders, and our findings are subject to nonresponse bias. Second, because recruitment was based on a combination of social media and Cedars-Sinai Medical Center physicians, selection bias is a concern. Third, radiation oncologists are overrepresented in our survey respondents, and the median age of respondents was 42 years, which may limit the generalizability of our findings. Fourth, we used a nonvalidated survey that may be subject to recall bias. In addition, while underrepresented minority groups in medicine have been shown to have a higher prevalence of burnout,^37^ we did not observe an association with TBB—we suspect due to the small sample size. Fifth, when collecting tumor board start times, given the typical absence of publicly reachable, centralized administrative staff, we were unable to verify start times independently, and they may have been subject to change since data collection.

## Conclusions

In this survey study of TBB, identifying as a woman or parent was independently associated with a higher level of burden from early or late tumor boards. Having 2 or more children, attending 3 or more hours per week of tumor boards, and radiology or pathology specialty were associated with a higher burden overall. Future strategies should aim to decrease burden, particularly the disparate burden on parents and women. These strategies should include organizational changes to improve work-life balance and the wellness of physicians and their families, increasing tumor board efficiency, and/or providing compensation for tumor board time.

## Notes

Supplement 1. **eFigure 1.** Word Clouds Summarizing the Most Frequently Encountered Respondent Comments Related to Early/Late Tumor Boards Negatively Affecting (A) Childcare (e.g., Daycare, Nanny) or (B) Child Feeding (Including Direct and/or Indirect Breastfeeding) and/or Sleep Logistics **eFigure 2.** Tumor Board Start Times From 22 NCI-Designated Cancer Centers and/or U.S. World and News Report Top 40 Hospitals for Cancer **eTable.** Alphabetical Listing of National Cancer Institute-Designated Cancer Centers and/or U.S. World and News Report Top 40 Hospitals for Cancer From Which Tumor Board Start Times Were Collected **eAppendix.** Survey: Administrative Burden of Tumor Boards

Supplement 2.
Data Sharing Statement
