# Supplementary material for: Association of Parental Status and Gender With Burden of Multidisciplinary Tumor Boards Among Oncology Physicians
Source: JAMA Netw Open. 2023 Oct 31;6(10):e2340663. doi: 10.1001/jamanetworkopen.2023.40663 (PMC10618838; doi:10.1001/jamanetworkopen.2023.40663)
Supplement: Supplement 1. — eFigure 1. Word Clouds Summarizing the Most Frequently Encountered Respondent Comments Related to Early/Late Tumor Boards Negatively Affecting (A) Childcare (e.g., Daycare, Nanny) or (B) Child Feeding (Including Direct and/or Indirect Breastfeeding) and/or Sleep Logistics eFigure 2. Tumor Board Start Times From 22 NCI-Designated Cancer Centers and/or U.S. World and News Report Top 40 Hospitals for Cancer eTable. Alphabetical Listing of National Cancer Institute-Designated Cancer Centers and/or U.S. World and News Report Top 40 Hospitals for Cancer From Which Tumor Board Start Times Were Collected eAppendix. Survey: Administrative Burden of Tumor Boards [file jamanetwopen-e2340663-s001.pdf]

Supplementary Online Content

Chau BL, LaGuardia JS, Kim S, et al. Association of parental status and gender with burden of multidisciplinary tumor boards among oncology physicians. *JAMA Netw Open.* 2023;6(10):e2340663. doi:10.1001/jamanetworkopen.2023.40663

eFigure 1. Word Clouds Summarizing the Most Frequently Encountered Respondent Comments Related to Early/Late Tumor Boards Negatively Affecting (A) Childcare (e.g., Daycare, Nanny) or (B) Child Feeding (Including Direct and/or Indirect Breastfeeding) and/or Sleep Logistics
eFigure 2. Tumor Board Start Times From 22 NCI-Designated Cancer Centers and/or U.S. World and News Report Top 40 Hospitals for Cancer
eTable. Alphabetical Listing of National Cancer Institute-Designated Cancer Centers and/or U.S. World and News Report Top 40 Hospitals for Cancer From Which Tumor Board Start Times Were Collected
eAppendix. Survey: Administrative Burden of Tumor Boards

This supplementary material has been provided by the authors to give readers additional information about their work.

© 2023 Chau BL et al. *JAMA Network Open.*

eFigure 1. Word Clouds Summarizing the Most Frequently Encountered Respondent Comments Related to Early/Late Tumor Boards Negatively Affecting (A) Childcare (e.g., Daycare, Nanny) or (B) Child Feeding (Including Direct and/or Indirect Breastfeeding) and/or Sleep Logistics

A. Childcare

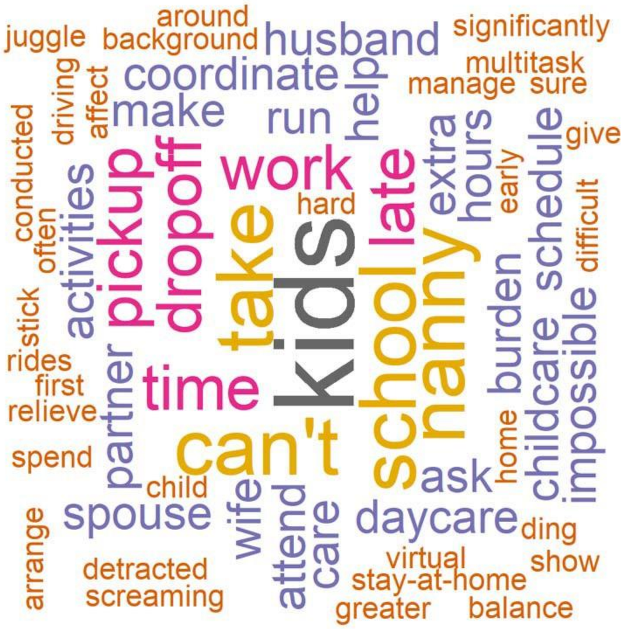

B. Child feeding and/or sleep logistics

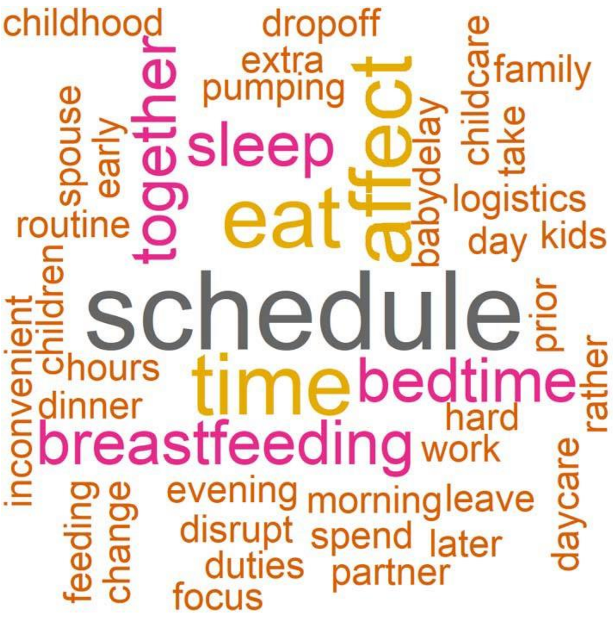

© 2023 Chau BL et al. *JAMA Network Open.*

eFigure 2. Tumor Board Start Times From 22 NCI-Designated Cancer Centers and/or U.S. World and News Report Top 40 Hospitals for Cancer. Start Times Were Designated From 0730 or Earlier to 1700 or Later in Half-Hour Increments. The Number of Tumor Boards is Depicted on the y-Axis With Respect to Start Times (x-Axis), With Percentages on Each Bar.

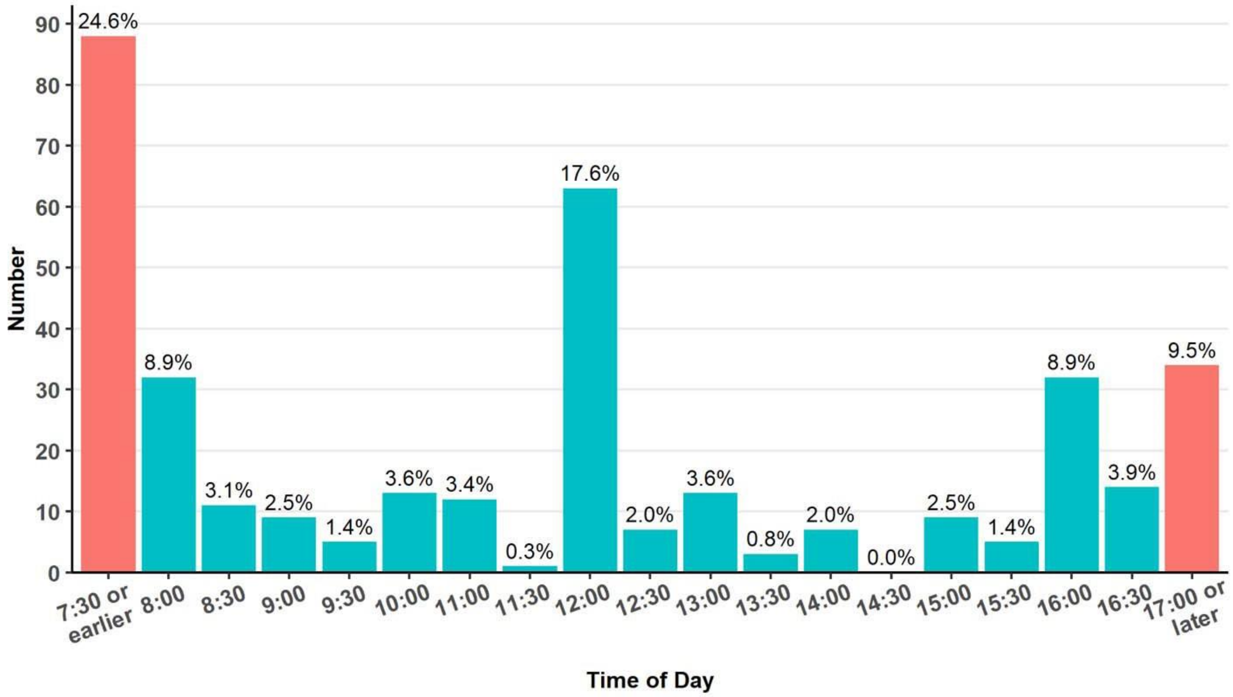

© 2023 Chau BL et al. *JAMA Network Open.*

eTable. Alphabetical Listing of National Cancer Institute-Designated Cancer Centers and/or U.S. World and News Report Top 40 Hospitals for Cancer From Which Tumor Board Start Times Were Collected

**Institution Name**
Brigham and Women's Hospital/Dana-Farber Cancer Institute
Cedars-Sinai Medical Center
City of Hope
Duke Cancer Institute
Emory Winship Cancer Center
Johns Hopkins - Sidney Kimmel Comprehensive Cancer Center
Massachusetts General Hospital
MD Anderson Cancer Center
Memorial Sloan Kettering Cancer Center
Ohio State University Comprehensive Cancer Center
Oregon Health & Science University
University of California Davis
University of California Los Angeles
University of California San Diego
University of North Carolina
University of Pittsburgh Medical Center
University of Southern California & Los Angeles County Medical Center
University of Texas Southwestern Medical Center
University of Washington
University of Alabama
University of California San Francisco
Washington University

© 2023 Chau BL et al. *JAMA Network Open.*

eAppendix. Survey: Administrative Burden of Tumor Boards

Dear Oncology Physicians,

We are asking you to complete an anonymous survey as part of a research study evaluating the administrative burden of multi-disciplinary tumor boards among U.S. oncology physicians. The Cedars-Sinai Principal Investigator is Dr. Katelyn Atkins, MD, PhD. The study contact phone number at Cedars-Sinai is 310-423-8077.

We are doing this study to measure the administrative burden of tumor boards among U.S. physicians involved in cancer care. The survey will include questions to find out if there are factors associated with increased administrative burden. We think this one-time survey should take about 5 minutes for you to complete. The survey is anonymous. It will have no information that could be used to identify you. We hope the information learned from this research study will help facilitate steps to reduce administrative burden and physician burnout.

Taking part in this research study is voluntary. You have the right to choose not to take part. You can also stop taking part at any time. You will not lose any services, benefits or rights you would normally have if you choose not to take part or stop taking part. Your choice not to take part or to stop taking part will not affect the care you get at Cedars-Sinai. You should not expect to benefit from taking part in this research study. You will not be paid for taking part in this research study.

If you would like to participate in the survey, please complete the survey below.

Thank you!

Katelyn Atkins, MD PhD

Department of Radiation Oncology

Cedars-Sinai Medical Center

Please contact the investigator at the phone number above for questions, problems, or concerns about the research. You might have feedback, questions, problems, concerns or want to obtain more information about this study. If so, you can talk with someone who is not part of this study by contacting:

Cedars-Sinai Human Research Protection Program (HRPP)

Phone: 310-423-3783

Email: ResearchConcerns@cshs.org

Website: cedars-sinai.org/research/administration/office-of-research-compliance/review-board.html

© 2023 Chau BL et al. *JAMA Network Open.*

Thinking about all the tumor boards you attend in a typical 30-day period, please answer the following questions.

© 2023 Chau BL et al. *JAMA Network Open.*

What is your specialty?

- ☐ Medical oncology
- ☐ Radiation oncology
- ☐ Surgical oncology
- ☐ Other surgical subspecialty
- ☐ Radiology
- ☐ Pathology
- ☐ Other

© 2023 Chau BL et al. *JAMA Network Open.*

What is your surgical specialty?	__________________________________
What is your specialty?	__________________________________
How many years have you been in practice?	◯ 0-5 years<br>◯ 6-10 years<br>◯ 11-15 years<br>◯ >15 years<br>◯ NA-currently a resident or fellow

© 2023 Chau BL et al. *JAMA Network Open.*

What type of institution do you work at? Check all that apply.

- ☐ Academic medical center
- ☐ Academic medical center affiliate or network site
- ☐ Community hospital
- ☐ Hospital-based practice
- ☐ Private or group practice
- ☐ NCI-designated comprehensive cancer center

© 2023 Chau BL et al. *JAMA Network Open.*

How many disease sites do you primarily treat (e.g., breast, GI, thoracic, GU, Gyn, CNS, etc)?	◯ 1-2<br>◯ 3-4<br>◯ 4+
How many total hours of tumor boards do you attend on an average week?	◯ 1-2 hours<br>◯ 3-4 hours<br>◯ 5-6 hours<br>◯ >6 hours<br>◯ Not applicable (0 hours)
How many hours of tumor boards are before 8am or after 5pm on an average week?	◯ 1-2 hours<br>◯ 3-4 hours<br>◯ 5-6 hours<br>◯ >6 hours<br>◯ Not applicable (0 hours)
How many additional hours are spent on other departmental or multi-disciplinary rounds (e.g., chart rounds, contour rounds) on an average week?	◯ 1-2 hours<br>◯ 3-4 hours<br>◯ 5-6 hours<br>◯ >6 hours<br>◯ Not applicable (0 hours)
How many hours are spent on other administrative work or in meetings on an average week?	◯ 1-2 hours<br>◯ 3-4 hours<br>◯ 5-6 hours<br>◯ >6 hours<br>◯ Not applicable (0 hours)

© 2023 Chau BL et al. *JAMA Network Open.*

Are you compensated for your time in tumor boards?	◯ Yes<br>◯ No
How are you compensated?	◯ RVU<br>◯ Monetary<br>◯ Other
Other compensation	__________________________________
Do you have children?	◯ Yes<br>◯ No

© 2023 Chau BL et al. *JAMA Network Open.*

How old are they? (check all that apply)

- ☐ 0-2 years
- ☐ 3-5 years
- ☐ 6-12 years
- ☐ 13+ years

© 2023 Chau BL et al. *JAMA Network Open.*

Do tumor boards before 8 am or after 5pm negatively affect your childcare (e.g., daycare, nanny) logistics?	◯ Yes<br>◯ No<br>◯ Not applicable
If yes, you may elaborate here (optional):	__________________________________
Do tumor boards before 8 am or after 5pm negatively affect your child/children's feeding (including direct and/or indirect breastfeeding) and/or sleep logistics?	◯ Yes<br>◯ No<br>◯ Not applicable
If yes, you may elaborate here (optional):	__________________________________
What percentage of your tumor boards are virtual because of the Covid-19 pandemic?	◯ < 25%<br>◯ 26%-50%<br>◯ 51%-75%<br>◯ 76%-100%

© 2023 Chau BL et al. *JAMA Network Open.*

Please rate the level of burden tumor boards present to you on a scale of 1-4.

Not at all burdensome
Slightly burdensome
Moderately burdensome
Very burdensome

© 2023 Chau BL et al. *JAMA Network Open.*

© 2023 Chau BL et al. *JAMA Network Open.*

◯
◯
◯
◯

Please rate the level of burden tumor boards before 8 am or after 5pm present to you on a scale of 1-4.

◯ Not at all burdensome
◯ Slightly burdensome
◯ Moderately burdensome
◯ Very burdensome ◯ Not applicable

© 2023 Chau BL et al. *JAMA Network Open.*

Please select the option which most closely reflects your level of agreement with each statement.

	Strongly disagree	Disagree	Neither agree nor disagree	Agree	Strongly agree	Not applicable or don't know
The amount of tumor boards I attend is burdensome.	○	○	○	○	○	○
The amount of tumor boards I attend contributes to my personal burnout.	○	○	○	○	○	○
Covid and the transition to virtual tumor boards has made attending tumor boards easier.	○	○	○	○	○	○
Covid and the transition to virtual meetings has increased my administrative burden.	○	○	○	○	○	○
I would prefer tumor boards to remain virtual in the future.	○	○	○	○	○	○
The early and/or late tumor boards negatively affect my childcare logistics.	○	○	○	○	○	○
The early and/or late tumor boards negatively affect my child/children's feeding (including direct or indirect breastfeeding) and/or sleep logistics.	○	○	○	○	○	○
The early and/or late tumor boards negatively affects my family dynamics.	○	○	○	○	○	○

© 2023 Chau BL et al. *JAMA Network Open.*

Your completion of the following demographic and background questions will greatly aid in the
analysis of the survey results.

What is your current age? ______________________

To which gender to you most identify?
- ◯ Transgender woman
- ◯ Woman
- ◯ Non-binary/non-conforming
- ◯ Transgender man
- ◯ Man
- ◯ Prefer not to say
- ◯ Prefer to self-describe

Prefer to self-describe (optional): ______________________

Are you Hispanic or Latinx/a/o/e (of Cuban, Mexican, Puerto Rican, South or Central American, or other Spanish culture or origin, regardless of race)?
- ◯ Yes
- ◯ No

© 2023 Chau BL et al. *JAMA Network Open.*

What race(s) do you identify as?

- ☐ American Indian or Alaska Native
- ☐ Asian
- ☐ Black or African American
- ☐ Native Hawaiian or Other Pacific Islander
- ☐ Middle Eastern or North African
- ☐ White
- ☐ Prefer not to say
- ☐ Prefer to self-describe

© 2023 Chau BL et al. *JAMA Network Open.*

Prefer to self-describe (optional):

____

Is there anything else you would like to share about administrative burden ?

____

Do you have any feedback about the survey?

____

© 2023 Chau BL et al. *JAMA Network Open.*
