# Supplementary material for: Association of Parental Status and Gender With Burden of Multidisciplinary Tumor Boards Among Oncology Physicians
Source: JAMA Netw Open. 2023 Oct 31;6(10):e2340663. doi: 10.1001/jamanetworkopen.2023.40663 (PMC10618838; doi:10.1001/jamanetworkopen.2023.40663)
Supplement: Supplement 2. — Data Sharing Statement [file jamanetwopen-e2340663-s002.pdf]

Data Sharing Statement

Chau. Association of Parental Status and Gender With Burden of Multidisciplinary Tumor Boards Among Oncology Physicians. *JAMA Netw Open*. Published October 31, 2023. doi:10.1001/jamanetworkopen.2023.40663

Data
Data available: No
